# Real-World Outcomes of First-Line Pembrolizumab-Based Therapy in Advanced Non-Small-Cell Lung Cancer: A Retrospective Single-Center Study

**DOI:** 10.3390/jcm15124757

**Published:** 2026-06-18

**Authors:** Einav Koren, Adar Yaacov, Jamal Zidan, Laila C. Roisman, Nir Peled, Noam Asna

**Affiliations:** 1 Clalit Health Services, Tel Aviv 6209804, Israel; 2 Helmsley Cancer Center, Shaare Zedek Medical Center, Jerusalem 9103102, Israel; 3 Faculty of Medicine, The Hebrew University of Jerusalem, Jerusalem 9112102, Israel; 4 The Oncology Department, Ziv Medical Center, and the Azrieli Faculty of Medicine, Bar-Ilan University, Zefat 1311502, Israel; 5 Cancer Research Institute, Samson Assuta Hospital, Ashdod 7747629, Israel; 6 Faculty of Health Sciences—Microbiology and Immunology, Ben Gurion University, Beer Sheva 8410501, Israel

**Keywords:** non-small-cell lung cancer, pembrolizumab, immunotherapy, real-world evidence, overall survival, progression-free survival, PD-L1, Cox regression

## Abstract

**Background:** Pembrolizumab-based therapy is a standard first-line option for advanced non-small-cell lung cancer (NSCLC), yet pivotal clinical-trial populations may not reflect patients encountered in routine practice. Real-world cohorts enriched for Eastern Cooperative Oncology Group performance status (ECOG PS) ≥2 and high metastatic burden remain underreported. We assessed real-world outcomes of first-line pembrolizumab in a heterogeneous cohort enriched for these. **Methods:** Retrospective cohort analysis of 45 patients with advanced NSCLC who received first-line pembrolizumab-based therapy (monotherapy or with platinum-based chemotherapy) at a single health maintenance organization in Israel between September 2017 and April 2020. **Results:** Mean age was 69.3 years (SD 9.0), 82.2% were male, 91.1% were current or former smokers, 37.8% had ECOG PS ≥2 (including 17.8% with ECOG ≥3), and 53.3% had three or more metastatic organ sites. PD-L1 expression was ≥50% in 46.7%, 1–49% in 13.3%, and <1% in 22.2%. After a median follow-up of 48.7 months (88.9% event rate), median overall survival (OS) was 8.87 months (95% CI, 5.88–14.32) and median progression-free survival (PFS) was 4.20 months (95% CI, 2.76–6.18), with an objective response rate of 46.7% and a disease control rate of 68.9%. On univariate Cox regression, the number of metastatic sites was most strongly associated with OS (HR 1.41 per site, 95% CI, 1.17–1.70, *p* = 0.0003). PD-L1 expression was significantly associated with both PFS (*p* < 0.0001) and OS (*p* = 0.0012), with the longest survival observed in patients with PD-L1 ≥50%. **Conclusions:** In this real-world cohort enriched for poor performance status and high metastatic burden, pembrolizumab-based therapy provided clinical benefit, but observed survival was substantially shorter than that reported in pivotal trials.

## 1. Introduction

Lung cancer is the leading cause of cancer-related mortality worldwide, accounting for approximately 1.8 million deaths annually [1]. Lung cancer is also a major contributor to cancer mortality in Israel, where incidence has continued to rise over recent decades [2]. Non-small-cell lung cancer (NSCLC) constitutes approximately 85% of all lung malignancies, and the prognosis for patients with advanced-stage disease remains poor, with 5-year overall survival rates of 0–10% for stage IV disease [3].

Immune checkpoint inhibitors have substantially changed the treatment landscape for advanced NSCLC. Pembrolizumab, a humanized monoclonal antibody targeting programmed death receptor 1 (PD-1), was among the first agents to demonstrate significant survival improvements in this population, initially in the phase Ib KEYNOTE-001 trial [4] and the phase III KEYNOTE-024 trial [5], paralleled by survival benefit with the anti–PD-1 nivolumab in CheckMate 057 [6]. KEYNOTE-024 established pembrolizumab as a first-line standard of care for patients with advanced NSCLC and PD-L1 tumor proportion scores (TPS) ≥50%. The updated analysis reported a median OS of 30.0 months and median PFS of 10.3 months [7], and long-term follow-up confirmed sustained benefit, with a 5-year OS rate of 31.9% [8]. The KEYNOTE-042 trial subsequently expanded the indication to patients with PD-L1 TPS ≥1%, reporting a median OS of 20.0 months [9].

However, the generalizability of randomized controlled trial results to everyday clinical practice has been increasingly questioned [10,11]. Trial enrollees are typically younger, fitter, and have less extensive disease than patients treated in routine practice. For example, patients ≥65 years of age and those of advanced age and frailty are markedly underrepresented in cancer treatment trials [12,13]. Real-world evidence (RWE) thus complements trial data by including older adults, patients with Eastern Cooperative Oncology Group Performance Status (ECOG PS) ≥2, and those with significant comorbidities [10,11]. Galán et al. reported that pembrolizumab outcomes in ECOG PS ≥2 patients are markedly worse than in fitter patients, a finding that cannot be derived from RCTs that exclude such patients [14].

Real-world data published to date have nevertheless shown limited coverage of the population subgroups most underrepresented in trials. Cohorts such as PEMBREIZH and that of Velcheti et al. predominantly enrolled patients with ECOG PS 0–1, with ECOG ≥2 representation typically below 15%, and few studies have systematically reported outcomes stratified by metastatic burden as a continuous variable. As a consequence, the magnitude of the survival gap from pivotal trials in patients with combined poor performance status, heavy metastatic burden, and advanced age—the patients most commonly encountered by clinicians outside academic centers—has remained imprecisely quantified.

The purpose of this study was to characterize the real-world outcomes of first-line pembrolizumab-based therapy in a single-center Israeli cohort enriched for ECOG PS ≥2 and high metastatic burden to identify clinical and pathological factors associated with overall and progression-free survival, and to contextualize these findings against pivotal trials and prior real-world cohorts.

## 2. Materials and Methods

### 2.1. Study Design and Population

This was a retrospective cohort study using medical records from the Meuhedet Health Maintenance Organization (HMO) in Israel. Eligible patients were adults (≥18 years) with histologically confirmed NSCLC, stage IIIB or IV as per the AJCC/UICC 8th edition TNM staging system, who received first-line pembrolizumab-based therapy (200 mg intravenously every 3 weeks) either as monotherapy or in combination with platinum-based chemotherapy between September 2017 and April 2020. Treatment regimen was selected at the treating physician’s discretion, based primarily on PD-L1 tumor proportion score: monotherapy was preferred for patients with PD-L1 ≥50%, and pembrolizumab plus platinum-based chemotherapy was preferred for patients with PD-L1 <50% who were judged fit for combination therapy. Comorbidity burden, ECOG performance status, and patient preference were considered as modifiers. Pembrolizumab monotherapy was also offered to selected patients with ECOG 3–4 in whom the treating physician judged that poor performance status was driven primarily by tumor burden likely to improve with disease response. Patients with incomplete medical records were excluded.

### 2.2. Data Collection and Variables

Variables abstracted from electronic medical records were: demographic characteristics (age at treatment initiation, sex); clinical variables (comorbidities, smoking status, baseline and on-treatment systemic corticosteroid use); oncologic parameters (histology, disease stage, presence of brain and liver metastases at treatment initiation, number of metastatic organ sites, ECOG PS, genomic alterations); PD-L1 tumor proportion score (assessed on tumor biopsy specimens by immunohistochemistry assay performed at central institutional laboratories); treatment outcomes (OS, PFS, and best overall response as per RECIST 1.1, classified as complete response [CR], partial response [PR], stable disease [SD], or progressive disease [PD]); and adverse events (AEs), including individual immune-related events and reasons for treatment discontinuation. Molecular testing was performed on tumor tissue for all 45 patients prior to pembrolizumab initiation. Adverse events were ascertained by chart review of physician progress notes, nursing notes, oncology day-unit records, and emergency department visits from the first pembrolizumab dose through the last documented follow-up (extended for immune-related events at chronic risk of relapse). All AEs documented in the medical record were captured as present/absent regardless of grade. CTCAE v5.0 grading was not consistently documented in the source records and was therefore not retrospectively assigned. Immune-related events (pneumonitis, colitis, hepatitis, endocrinopathies) were physician-adjudicated at the time of the clinical event and were not re-adjudicated for this analysis.

### 2.3. Statistical Analysis

Continuous variables are summarized as means ± standard deviation (SD) or medians with interquartile ranges (IQRs) and categorical variables as frequencies and percentages. OS was measured from pembrolizumab initiation to death from any cause. Patients alive at last follow-up were censored. PFS was measured to disease progression or death, whichever came first [15,16]. Median follow-up was estimated by the reverse Kaplan–Meier method [17]. Survival curves were compared by the log-rank test. Univariate Cox proportional-hazard regression was used to estimate hazard ratios (HRs) and 95% confidence intervals (CIs), and variables with *p* <0.1 plus clinically important factors (age, ECOG PS) were entered into a multivariate model. The proportional-hazard assumption was tested by Schoenfeld residuals [18]. Adverse events were recorded as binary (present/absent). CTCAE grading was unavailable. A two-sided *p* < 0.05 was considered significant. Analyses used Python 3.12 with lifelines 0.30.0 and SciPy 1.15.3. A sensitivity analysis was performed by repeating the Kaplan–Meier and univariate Cox analyses in the subgroup restricted to patients with ECOG performance status 0–2. Baseline characteristics were compared between monotherapy and combination-therapy regimen groups using Welch’s *t*-test for continuous variables and Fisher’s exact test or chi-squared test for categorical variables (Supplementary Table S1).

### 2.4. Ethical Considerations

All patient data were deidentified prior to analysis. Access to identifiable patient information was restricted to the research team and maintained within Meuhedet’s secure systems. The study was approved by the Meuhedet Ethics Committee on 22 August 2022 (study 08-31-08-22). The requirement for informed consent was waived given the retrospective design.

During the preparation of this manuscript, the authors used Claude Opus, versions 4.1, 4.5 and 4.6 for the purposes of supporting the analyses and writing process. The authors have reviewed and edited the output and take full responsibility for the content of this publication.

## 3. Results

### 3.1. Patient Characteristics

A total of 45 patients with advanced NSCLC who received first-line pembrolizumab-based therapy were identified (Table 1 and Table 2). Baseline characteristics by treatment regimen (monotherapy vs. combination therapy) are presented in Supplementary Table S1. The two groups were well matched on most covariates, but differed markedly in PD-L1 distribution (≥50% in 93.3% of monotherapy patients vs. 33.3% of combination patients; Fisher’s exact *p* = 0.001), as PD-L1 status is the principal driver of regimen selection in routine practice. Of the 45 patients, 18 (40.0%) required systemic corticosteroid treatment during the course of pembrolizumab-based therapy.

### 3.2. Survival Outcomes

**Figure 1:**
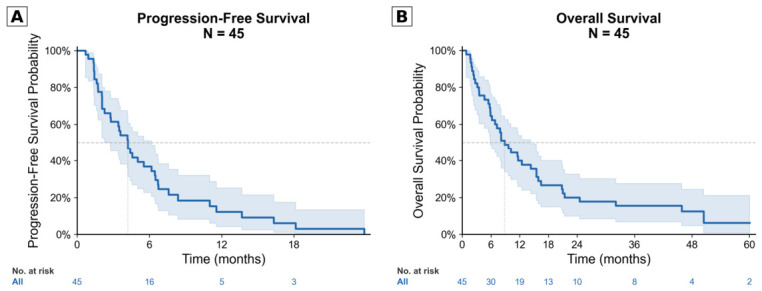
Kaplan–Meier estimates for the full cohort (N = 45). (**A**) Progression-free survival; (**B**) overall survival. shaded area = 95% Confidence Interval.

Median follow-up was 48.7 months. At data cutoff, 40 patients (88.9%) had died and 5 (11.1%) were alive. Median PFS was 4.20 months (95% CI, 2.76–6.18) and median OS was 8.87 months (95% CI, 5.88–14.32) (Figure 1). The 6-, 12-, and 24-month OS rates were 64.4%, 40.0%, and 20.0%, respectively. Corresponding PFS rates were 36.8%, 12.3%, and 0.0%.

### 3.3. Treatment Response

As per RECIST 1.1: 4 patients (8.9%) achieved CR, 17 (37.8%) PR, 10 (22.2%) SD, 10 (22.2%) PD. One patient (2.2%) had progressive brain disease with stable systemic disease, and tumor response was not assessable in three (6.7%). Overall response rate was 46.7% and the disease control rate was 68.9% (Table 3).

### 3.4. Univariate and Multivariate Survival Analyses

The number of metastatic sites (continuous: HR 1.41 per site, 95% CI 1.17–1.70, *p* = 0.0003; ≥3 vs. <3: HR 2.72, 95% CI 1.42–5.23, *p* = 0.003) and prior surgical history (HR 0.42, 95% CI 0.18–0.95, *p* = 0.037) were significantly associated with OS. For PFS, PD-L1 ≥50% vs. <50% (HR 0.40, *p* = 0.026), number of metastatic sites (HR 1.32, *p* = 0.013), and liver metastases (HR 2.53, *p* = 0.051) showed significant or borderline-significant associations (Table 4).

Multivariate Cox regression for OS included age, ECOG PS ≥2, liver metastases, three or more metastatic sites, and prior surgery (Table 5). Having three or more metastatic sites showed a trend toward worse OS (HR 2.07, 95% CI 0.99–4.35; *p* = 0.053), but no covariate reached *p* < 0.05 in the multivariate model, likely reflecting limited statistical power with 45 patients and 40 events. The Schoenfeld test indicated possible non-proportional hazards for ECOG PS (*p* = 0.04), suggesting that its effect on survival may vary over time. The concordance index was 0.69. In the sensitivity analysis restricted to patients with ECOG performance status 0–2 (n = 37, 32 deaths), median OS was 9.63 months (95% CI, 6.21–15.87) and median PFS was 4.40 months (95% CI, 3.42–6.44), only modestly higher than in the full cohort, indicating that the survival gap from pivotal-trial outcomes is not primarily attributable to inclusion of ECOG 3–4 patients (Supplementary Figure S1). The dominant prognostic role of metastatic burden persisted in this subgroup (HR 1.35 per site for OS, 95% CI, 1.10–1.66, *p* = 0.004; HR 2.48 for three or more vs. fewer than three sites, 95% CI, 1.20–5.13, *p* = 0.014; Supplementary Tables S2 and S3).

### 3.5. Prognostic Factors

**Figure 2:**
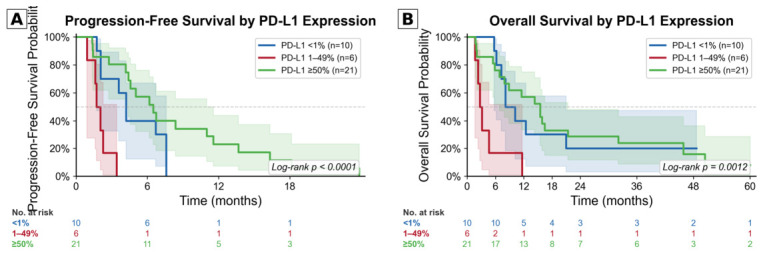
Survival by PD-L1 tumor proportion score. (**A**) Progression-free survival; (**B**) overall survival.

PD-L1 data were available for 37 patients (82.2%). Median PFS for PD-L1 <1%, 1–49%, and ≥50% was 4.20 (95% CI, 1.74–7.59), 2.04 (0.92–3.42), and 6.50 (4.17–11.56) months, respectively (log-rank *p* < 0.0001). Corresponding median OS was 8.11 (5.72–20.99), 3.12 (1.68–11.63), and 15.47 (6.87–21.39) months (*p* = 0.0012) (Figure 2).

**Figure 3:**
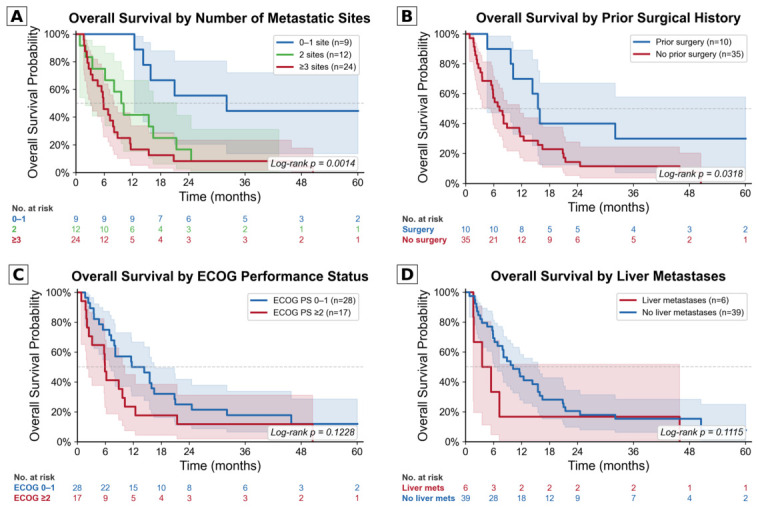
Overall survival by clinical prognostic factors. (**A**) Number of metastatic sites; (**B**) prior surgery for early-stage NSCLC; (**C**) ECOG performance status; (**D**) liver metastases at treatment initiation.

Mean number of metastatic organ sites was 2.93 ± 1.67. Median OS for patients with zero to one, two, and three or more metastatic sites was 32.06 (12.45–NR), 9.63 (2.04–21.39), and 5.85 (3.12–8.11) months, respectively (log-rank *p* = 0.001) (Figure 3A). Ten patients (22.2%) had a history of early-stage NSCLC treated by surgical resection prior to developing metastatic disease and showed significantly better OS than those without prior surgery (median 15.51 [4.63–NR] vs. 7.23 [5.45–11.53] months; *p* = 0.032) (Figure 3B). Patients with ECOG PS 0–1 (n = 28) had numerically longer median OS than those with ECOG PS ≥2 (n = 17: 11.63 [7.23–16.46] vs. 5.88 [2.04–10.18] months; *p* = 0.12) (Figure 3C). Six patients (13.3%) had liver metastases at treatment initiation. Median OS was numerically lower in this subgroup (5.45 [1.68–45.86] vs. 10.18 [6.21–15.51] months; *p* = 0.11) (Figure 3D).

In sum, 17 patients (37.8%) received pembrolizumab monotherapy and 27 (60.0%) received pembrolizumab plus platinum-based chemotherapy. One regimen was undocumented. Patients on monotherapy had numerically longer median OS than those on combination therapy (14.32 months, 95% CI, 6.87–21.39 vs. 6.21 months, 95% CI, 3.52–11.63; log-rank *p* = 0.24). OS at 6, 12, and 24 months was 82.4%, 52.9%, and 23.5% for monotherapy vs. 51.9%, 29.6%, and 14.8% for combination. By individual chemotherapy partner, median OS was 14.32 months for pembrolizumab alone, 7.23 (4.63–15.51) months for pemetrexed/carboplatin, and 5.72 (2.04–12.45) months for paclitaxel/carboplatin (multi-group log-rank *p* = 0.21). This descriptive comparison is heavily confounded by indication: 93.3% of monotherapy patients had PD-L1 ≥50% compared with only 33.3% of combination patients (Supplementary Table S1; Fisher’s exact *p* = 0.001).

### 3.6. Adverse Events

The most commonly reported event was weakness/fatigue (44.4%). By organ system, gastrointestinal events were most frequent (26.7%), followed by pulmonary (15.6%) and dermatologic (6.7%). Specific immune-related events included diarrhea (11.1%), pneumonitis (11.1%), colitis (6.7%), and elevated transaminases (4.4%). A total of 16 patients (35.6%) permanently discontinued pembrolizumab because of toxicity, and 18 (40.0%) required systemic steroid treatment (Table 6). Patients who discontinued for adverse events had a longer median OS than those who did not (15.47 [5.85–20.99] vs. 8.11 [4.63–11.63] months). This descriptive comparison is potentially affected by immortal time bias and is interpreted further in the Discussion.

### 3.7. Comparison with Clinical Trials and Real-World Studies

Survival outcomes for the present study, two pivotal RCTs, and two prior real-world cohorts are summarized in Table 7.

## 4. Discussion

In this single-center retrospective cohort of 45 patients with advanced NSCLC followed for a median of 48.7 months, first-line pembrolizumab-based therapy provided clinical benefit, but observed survival was substantially shorter than that reported in pivotal trials. Median OS was 8.87 months and median PFS was 4.20 months, lower than KEYNOTE-024 (30.0 and 10.3 months) [7] and KEYNOTE-042 (20.0 and 7.1 months) [9] and below other real-world cohorts such as PEMBREIZH (15.2/10.1 months) [19] and Velcheti et al. (19.6/7.3 months) [20] (Table 7).

This report contributes to the growing real-world literature on first-line pembrolizumab in advanced NSCLC in several respects. First, the cohort is enriched for patients with ECOG performance status ≥2 (37.8%), including eight patients (17.8%) with ECOG 3–4—a population effectively absent from pivotal trials and less abundant from most prior real-world cohorts. Second, metastatic burden is examined as a continuous prognostic variable with mature follow-up (48.7 months, 88.9% event rate), allowing a more precise per-site hazard estimate (HR 1.41, 95% CI, 1.17–1.70, *p* = 0.0003) than is generally available from prior studies. The independent prognostic contributions of metastatic burden and functional-status trajectories are examined in greater depth [21].

This gap likely reflects the composition of our cohort, which differs from pivotal-trial populations in several ways. First, 37.8% of patients had ECOG PS ≥2 (including eight with ECOG ≥3)—a group systematically excluded from pivotal trials and associated with poorer outcomes in real-world studies [14]. Second, 53.3% of patients had three or more metastatic organ sites. The number of metastatic sites was the variable most strongly associated with OS on univariate analysis (HR 1.41 per site, *p* = 0.0003) and showed the strongest trend on multivariate analysis (HR 2.07, 95% CI 0.99–4.35; *p* = 0.053), although it did not reach conventional statistical significance. Third, our patients were older than typical trial enrollees (mean 69.3 vs. 64.5 years in KEYNOTE-024) [7].

PD-L1 expression was strongly associated with both PFS (*p* < 0.0001) and OS (*p* = 0.0012). The longest survival was observed in the PD-L1 ≥50% subgroup (median OS 15.47 months), consistent with prior reports [22,23]. An apparent dip in the 1–49% subgroup (median OS 3.12 months) almost certainly reflects its very few subjects (n = 6) rather than a true biological effect, and the result should be interpreted with caution. Heterogeneity in outcomes for PD-L1 <50% has also been reported in the PEOPLE trial [24].

The role of pembrolizumab in combination with platinum-based chemotherapy as a first-line regimen for advanced NSCLC is supported by two pivotal phase III trials. KEYNOTE-189 [25] established the benefit of pembrolizumab with pemetrexed and platinum in non-squamous NSCLC, with a 5-year overall survival update [26] showing sustained survival improvement over chemotherapy alone. KEYNOTE-407 [27] demonstrated parallel benefit in squamous NSCLC for pembrolizumab combined with carboplatin and paclitaxel or nab-paclitaxel, with sustained 5-year benefit subsequently confirmed [28] In our cohort, 27 of 45 patients received pembrolizumab plus platinum-based chemotherapy, and outcomes in this subgroup were numerically shorter than those reported in trial populations. This difference is consistent with the compositional differences described above and does not constitute evidence against the combination regimens themselves. A recent multicenter real-world study of first-line pembrolizumab monotherapy from Vietnam (Pham et al., 2026) [29] reported a median OS of 25.4 months in a cohort that—in contrast to ours—was enriched for PD-L1 ≥50% (86.3%) and ECOG PS 0–1 (75.3%), highlighting how differences in cohort composition translate into substantially different outcome profiles in real-world pembrolizumab series.

Liver metastases trended toward worse outcomes (univariate OS HR 2.01, *p* = 0.12; PFS HR 2.53, *p* = 0.051), consistent with prior literature identifying hepatic involvement as a negative prognostic factor for anti-PD-1 therapy in NSCLC [30,31]. Patients with prior surgery for early-stage NSCLC had longer OS (15.51 vs. 7.23 months, *p* = 0.032), an effect that did not persist on multivariate analysis (HR 0.77, *p* = 0.57) and likely reflects favorable disease biology and lead-time bias rather than a direct surgical benefit.

Treatment was discontinued for toxicity in 35.6% of patients and steroid treatment was required in 40.0%, underscoring the need for careful monitoring of immune-related toxicities in routine practice. Patients who discontinued for adverse events had a longer median OS than those who did not (15.47 vs. 8.11 months). A similar association between immune-related adverse events and efficacy has been described in NSCLC [32,33]. This finding should be interpreted with caution, however: as a post-baseline event, treatment discontinuation due to adverse events is susceptible to immortal time bias, since patients must remain alive long enough on therapy to develop a treatment-discontinuing adverse event.

This study has several limitations. The retrospective single-center design and the modest sample size limit multivariate power and likely account for the lack of independently significant covariates. The absence of a control arm precludes formal effectiveness comparisons. Restriction to a single Israeli HMO may limit external validity. PD-L1 expression was unavailable for eight patients (17.8%), and adverse-event data were captured as present/absent without CTCAE grading. The Schoenfeld test indicated possible non-proportional hazards for ECOG PS, suggesting that its effect on survival may vary over time. The monotherapy-versus-combination comparison is confounded by indication and was performed post hoc. The PD-L1 1–49% subgroup contained only six patients, so any inference about that stratum is unreliable. Finally, the association between treatment discontinuation due to adverse events and longer survival is potentially affected by immortal time bias and should be regarded as hypothesis-generating.

## 5. Conclusions

In a real-world Israeli cohort enriched for patients with poor performance status and high metastatic burden, first-line pembrolizumab-based therapy provided clinical benefit, but observed survival was substantially shorter than that reported in pivotal trials. Metastatic burden was the dominant prognostic factor. These results support the continued need for larger multicenter real-world studies—and prospective trials in poor-PS/high-burden populations—to refine outcome prediction, optimize regimen selection, and identify subgroups for whom alternative or combination therapy may yield additional benefit [34].

## Figures and Tables

**Table 1:** Baseline demographic and comorbidity characteristics (N = 45).

Characteristic	N (%)
**Age at treatment initiation, mean (SD), years**	69.3 (9.0)
Age ≥ 75	12 (26.7)
**Sex**	
Male	37 (82.2)
Female	8 (17.8)
**Smoking status**	
Current/former	41 (91.1)
Never	4 (8.9)
**ECOG PS at treatment initiation**	
0	15 (33.3)
1	13 (28.9)
2	9 (20.0)
3	7 (15.6)
4	1 (2.2)
0–1	28 (62.2)
≥2	17 (37.8)
**Comorbidities**	
Hypertension	18 (40.0)
Diabetes mellitus	12 (26.7)
COPD	9 (20.0)
Ischemic heart disease	8 (17.8)
**Steroid use during pembrolizumab**	18 (40.0)

ECOG PS, Eastern Cooperative Oncology Group Performance Status; SD, standard deviation.

**Table 2:** Disease characteristics and treatment regimen (N = 45).

Characteristic	N (%)
**Histology**	
Adenocarcinoma	30 (66.7)
Squamous	11 (24.4)
NSCLC NOS	4 (8.9)
**PD-L1 expression**	
<1%	10 (22.2)
1–49%	6 (13.3)
≥50%	21 (46.7)
Unknown	8 (17.8)
**Metastatic sites at treatment initiation**	
Brain	11 (24.4)
Liver	6 (13.3)
Bone	21 (46.7)
Lung (contralateral)	20 (44.4)
Pleura	15 (33.3)
Lymph nodes	29 (64.4)
Adrenal	9 (20.0)
**Number of metastatic organ sites**	
0–1	9 (20.0)
2	12 (26.7)
≥3	24 (53.3)
Mean (SD)	2.93 (1.67)
Prior surgery for early-stage NSCLC	10 (22.2)
**Molecular profile, n positive/tested (%)**	
EGFR mutation	3/45 (6.7)
KRAS (any)	6/45 (13.3)
BRAF mutation	2/45 (4.4)
ALK rearrangement	0/45 (0.0)
STK11 mutation	4/45 (8.9)
**Treatment regimen**	
Pembrolizumab monotherapy	17 (37.8)
Pembrolizumab + pemetrexed + carboplatin	16 (35.6)
Pembrolizumab + paclitaxel + carboplatin	10 (22.2)
Pembrolizumab + pemetrexed + cisplatin	1 (2.2)
Not documented	1 (2.2)
Number of cycles, median (IQR)	6 (3–9)

NOS, not otherwise specified; PD-L1, programmed death-ligand 1.

**Table 3:** Best overall response as per RECIST 1.1 (N = 45).

Response Category	N (%)
Complete response (CR)	4 (8.9)
Partial response (PR)	17 (37.8)
Stable disease (SD)	10 (22.2)
Progressive disease (PD)	10 (22.2)
Brain PD with systemic SD	1 (2.2)
Not assessed	3 (6.7)
Overall response rate (CR + PR)	21 (46.7)
Disease control rate (CR + PR + SD)	31 (68.9)

RECIST, Response Evaluation Criteria in Solid Tumors.

**Table 4:** Univariate Cox proportional-hazard regression for OS and PFS.

Variable	OS HR (95% CI)	OS p	PFS HR (95% CI)	PFS p
Age (continuous, per year)	0.98 (0.95–1.01)	0.24	0.98 (0.94–1.01)	0.15
Age ≥75 vs. <75	0.59 (0.29–1.22)	0.15	0.52 (0.24–1.11)	0.09
Sex (male vs. female)	1.13 (0.50–2.58)	0.76	1.44 (0.63–3.29)	0.39
ECOG PS ≥2 vs. 0–1	1.64 (0.87–3.12)	0.13	1.12 (0.57–2.17)	0.75
Smoking (ever vs. never)	0.93 (0.33–2.62)	0.89	0.57 (0.20–1.66)	0.31
Histology (squamous vs. adeno)	1.00 (0.47–2.12)	1.00	1.26 (0.59–2.68)	0.55
PD-L1 ≥50% vs. <50%	0.61 (0.30–1.26)	0.18	0.40 (0.18–0.89)	0.03
No. of met sites (continuous)	1.41 (1.17–1.70)	0.0003	1.32 (1.06–1.64)	0.01
Met sites ≥3 vs. <3	2.72 (1.42–5.23)	0.003	1.62 (0.85–3.10)	0.14
Brain metastases (yes vs. no)	0.95 (0.46–1.95)	0.89	0.96 (0.46–1.99)	0.91
Liver metastases (yes vs. no)	2.01 (0.84–4.85)	0.12	2.53 (1.00–6.43)	0.05
Prior surgery (yes vs. no)	0.42 (0.18–0.95)	0.04	0.47 (0.20–1.07)	0.07
Discontinuation due to AE	0.66 (0.34–1.28)	0.22	1.09 (0.56–2.16)	0.79

AE, adverse event; CI, confidence interval; HR, hazard ratio. The “discontinuation due to AE” covariate reflects a post-baseline event and is provided for descriptive purposes only. This estimate is potentially affected by immortal time bias and should not be interpreted as a causal effect on survival.

**Table 5:** Multivariate Cox proportional-hazard regression for OS (N = 44).

Variable	HR	95% CI	*p*
Age (continuous, per year)	0.99	0.95–1.02	0.52
ECOG PS ≥2 vs. 0–1	1.59	0.77–3.29	0.21
Liver metastases (yes vs. no)	1.26	0.44–3.56	0.67
Metastatic sites ≥3 vs. <3	2.07	0.99–4.35	0.053
Prior surgery (yes vs. no)	0.77	0.31–1.92	0.57

Model: N = 44, events = 40. Concordance = 0.69. Likelihood-ratio test *p* = 0.06.

**Table 6:** Adverse events during pembrolizumab treatment (N = 45).

Adverse Event	N (%)
**Individual adverse events**	
Weakness/fatigue	20 (44.4)
Diarrhea	5 (11.1)
Pneumonitis	5 (11.1)
Anorexia	4 (8.9)
Nausea/vomiting	3 (6.7)
Abdominal pain	3 (6.7)
Colitis	3 (6.7)
Pruritus	2 (4.4)
Elevated transaminases	2 (4.4)
Pain	2 (4.4)
Cough	2 (4.4)
Hypothyroidism	1 (2.2)
Arthralgia	1 (2.2)
Rash	1 (2.2)
Constipation	1 (2.2)
Encephalitis	1 (2.2)
Ptosis	1 (2.2)
**By organ system**	
Constitutional (weakness/pain)	21 (46.7)
Gastrointestinal	12 (26.7)
Pulmonary	7 (15.6)
Dermatologic	3 (6.7)
Hepatic	2 (4.4)
Neurologic	2 (4.4)
Endocrine	1 (2.2)
Musculoskeletal	1 (2.2)
**Summary**	
Any specific AE (excluding weakness)	24 (53.3)
Treatment discontinuation due to AE	16 (35.6)
Steroid treatment required	18 (40.0)

CTCAE grading was unavailable; AEs are reported as present/absent. AE, adverse event; CTCAE, Common Terminology Criteria for Adverse Events.

**Table 7:** Comparison of survival outcomes across clinical trials and real-world studies of first-line pembrolizumab in advanced NSCLC.

Study	Median PFS, Months (95% CI)	Median OS, Months (95% CI)	Type
Current study	4.20 (2.76–6.18)	8.87 (5.88–14.32)	RW
PEMBREIZH [19]	10.1 (8.8–NA)	15.2 (13.9–NA)	RW
Velcheti et al. [20]	7.3 (5.7–9.2)	19.6 (16.6–24.3)	RW
KEYNOTE-024 [7]	10.3 (6.7–NR)	30.0 (18.3–NR)	RCT
KEYNOTE-042 [9]	7.1 (5.9–9.0)	20.0 (15.9–24.2)	RCT

CI, confidence interval; NA, not available; NR, not reached; OS, overall survival; PFS, progression-free survival; RCT, randomized controlled trial; RW, real-world.

## Data Availability

The data presented in this study are available on request from the corresponding author. The data are not publicly available due to patient-privacy considerations.

## Supplementary Materials

The following supporting information can be downloaded at https://www.mdpi.com/article/10.3390/jcm15124757/s1. Table S1: Baseline characteristics by treatment regimen, Table S2: Median OS/PFS in the ECOG 0–2 sensitivity cohort, Table S3: Univariate Cox analyses in the ECOG 0–2 sensitivity cohort, Figure S1: Kaplan–Meier curves for the ECOG 0–2 sensitivity cohort vs. the full cohort, Figure S2: Best overall response per RECIST v1.1.
