# Supplementary material for: Real-World Outcomes of First-Line Pembrolizumab-Based Therapy in Advanced Non-Small-Cell Lung Cancer: A Retrospective Single-Center Study
Source: J Clin Med. 2026 Jun 18;15(12):4757. doi: 10.3390/jcm15124757 (PMC13302034; doi:10.3390/jcm15124757)
Supplement: Supplementary file 1 [file jcm-15-04757-s001.zip › jcm-4317050-supplementary.pdf]

Supplementary Materials

Real-World Outcomes of First-Line Pembrolizumab-Based Therapy in Advanced Non-Small Cell Lung Cancer: A Retrospective Single-Center Study

Table S1. Baseline characteristics by first-line treatment regimen (monotherapy vs combination chemo-immunotherapy).

Variable	Monotherapy (n=17)	Combination (n=27)	p-value	Test
**Age at treatment start, years (mean ± SD)**	69.4 (10.5)	69.0 (8.3)	0.894	Welch's t
**Age ≥75 years**	5 (29.4%)	6 (22.2%)	0.724	Fisher
**Male sex**	15 (88.2%)	21 (77.8%)	0.455	Fisher
**Ever smoker**	14 (82.4%)	26 (96.3%)	0.282	Fisher
**ECOG PS ≥2**	6 (35.3%)	10 (37.0%)	1.0	Fisher
**Histology**	Adenocarcinoma 14 (82.4%); Squamous 2 (11.8%); NSCLC NOS 1 (5.9%)	Adenocarcinoma 16 (59.3%); Squamous 8 (29.6%); NSCLC NOS 3 (11.1%)	0.273	Chi-square
**PD-L1 category**	<1% 1 (6.7%); 1-49% 0 (0.0%); >=50% 14 (93.3%)	<1% 8 (38.1%); 1-49% 6 (28.6%); >=50% 7 (33.3%)	0.001	Chi-square
**Number of metastatic sites (mean ± SD)**	2.9 (1.5)	3.0 (1.7)	0.846	Welch's t
**≥3 metastatic sites**	9 (52.9%)	15 (55.6%)	1.0	Fisher
**Brain metastases (at IO start)**	6 (35.3%)	5 (18.5%)	0.289	Fisher
**Liver metastases (at IO start)**	2 (11.8%)	4 (14.8%)	1.0	Fisher
**Prior surgery for early-stage NSCLC**	3 (17.6%)	7 (25.9%)	0.716	Fisher
**EGFR mutation**	3 (17.6%)	0 (0.0%)	0.051	Fisher
**KRAS mutation (any)**	1 (5.9%)	5 (18.5%)	0.38	Fisher
**STK11 mutation**	0 (0.0%)	4 (14.8%)	0.147	Fisher
**Hypertension**	7 (41.2%)	11 (40.7%)	1.0	Fisher
**Diabetes mellitus**	6 (35.3%)	6 (22.2%)	0.489	Fisher
**COPD**	1 (5.9%)	7 (25.9%)	0.125	Fisher
**Ischemic heart disease**	6 (35.3%)	2 (7.4%)	0.04	Fisher
**Number of cycles administered (mean ± SD)**	10.6 (8.7)	5.3 (2.5)	0.025	Welch's t

Continuous variables compared by Welch's t-test; categorical variables by Fisher's exact test (2×2) or chi-square test. One patient with an undocumented regimen is excluded. ECOG PS, Eastern Cooperative Oncology Group performance status; PD-L1, programmed death-ligand 1; SD, standard deviation.

Table S2. Median overall and progression-free survival: full cohort vs ECOG 0-2 sensitivity cohort.

Cohort	N	Deaths	Median OS (months)	Median PFS (months)
**Full cohort**	45	40	8.87 (5.88–14.32)	4.20 (2.76–6.18)
**ECOG 0-2 only**	37	32	9.63 (6.21–15.87)	4.40 (3.42–6.44)

Eight patients with ECOG performance status 3-4 (seven ECOG 3 and one ECOG 4) were excluded for the sensitivity analysis. CI, confidence interval; OS, overall survival; PFS, progression-free survival.

Table S3. Univariate Cox proportional-hazards regression for OS and PFS in the ECOG 0-2 sensitivity cohort.

Variable	Endpoint	HR (95% CI)	p
**Age (per year)**	OS	0.97 (0.94–1.01)	0.168
**Age (per year)**	PFS	0.95 (0.91–0.99)	0.015
**ECOG PS ≥2 vs 0–1**	OS	1.40 (0.62–3.16)	0.424
**ECOG PS ≥2 vs 0–1**	PFS	0.90 (0.38–2.10)	0.803
**PD-L1 ≥50% vs <50%**	OS	0.57 (0.26–1.29)	0.181
**PD-L1 ≥50% vs <50%**	PFS	0.32 (0.13–0.80)	0.016
**No. metastatic sites (continuous)**	OS	1.35 (1.10–1.66)	0.004
**No. metastatic sites (continuous)**	PFS	1.25 (0.99–1.59)	0.065
**≥3 metastatic sites vs <3**	OS	2.48 (1.20–5.13)	0.014
**≥3 metastatic sites vs <3**	PFS	1.35 (0.65–2.77)	0.418
**Brain metastases**	OS	0.87 (0.37–2.02)	0.739
**Brain metastases**	PFS	1.14 (0.48–2.66)	0.77
**Liver metastases**	OS	1.87 (0.71–4.91)	0.206
**Liver metastases**	PFS	2.74 (0.96–7.78)	0.059
**Prior surgery**	OS	0.41 (0.17–1.01)	0.054
**Prior surgery**	PFS	0.44 (0.18–1.09)	0.076

Univariate Cox models fit within the ECOG 0-2 sensitivity cohort (n = 37). CI, confidence interval; HR, hazard ratio; OS, overall survival; PFS, progression-free survival.

Figure S1. Kaplan-Meier curves: ECOG 0-2 sensitivity cohort vs full cohort.

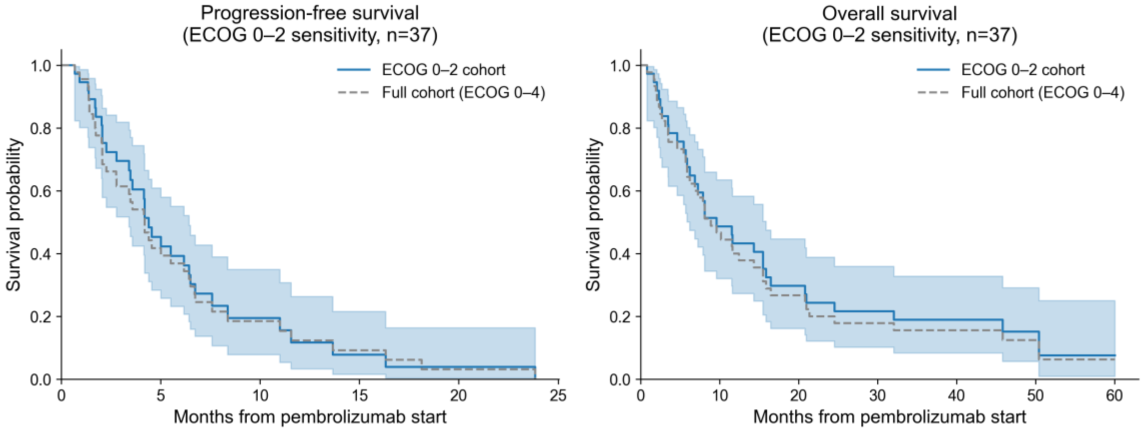

Figure S1. Kaplan-Meier estimates of progression-free survival (left) and overall survival (right) for the ECOG 0-2 sensitivity cohort (n = 37, solid blue) compared against the full cohort (n = 45, dashed grey). The eight patients with ECOG 3-4 (seven ECOG 3 and one ECOG 4) are excluded from the sensitivity cohort. The shaded ribbon shows the 95% pointwise confidence interval for the sensitivity cohort.

Figure S2. Best overall response per RECIST v1.1.

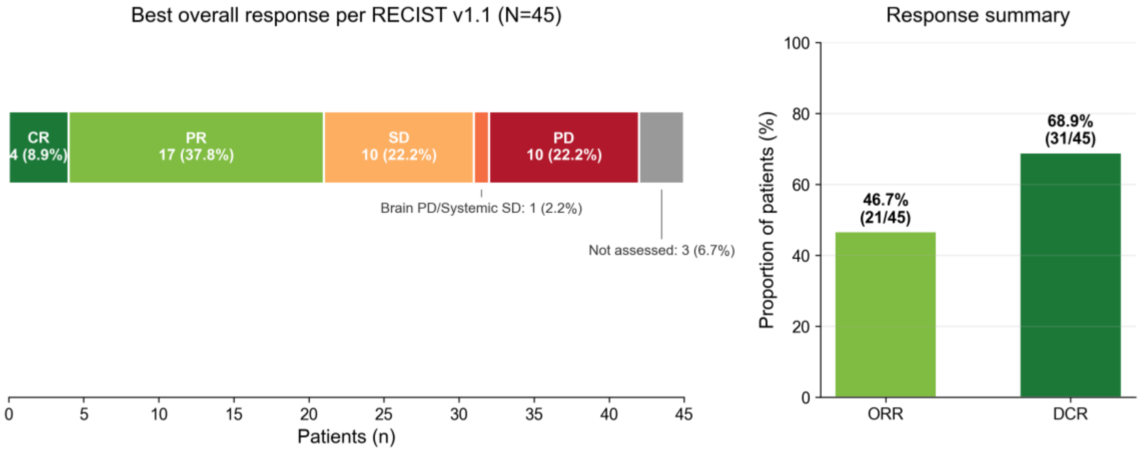

Figure S2. Best overall response per RECIST v1.1 for the full cohort (N = 45). Left: stacked horizontal bar showing the distribution of complete response (CR), partial response (PR), stable disease (SD), progressive disease (PD), brain progression with systemic stable disease, and not-assessed patients. Right: summary of objective response rate (ORR, CR + PR = 46.7%) and disease control rate (DCR, CR + PR + SD = 68.9%).
